# MBNL1 Promotes Intestinal Fibrosis via RAS-MAPK Pathway-Mediated Fibroblast Activation and Proliferation

**DOI:** 10.3390/biomedicines14061207

**Published:** 2026-05-27

**Authors:** Liwen Zhang, Tianqi Liu, Na Yu, Ruijian Zhang, Zhepeng Luo, Xiaoqing Zhang, Jiani Wang

**Affiliations:** 1 The First Clinical College, China Medical University, Shenyang 110001, China; 2023120642@cmu.edu.cn (L.Z.); tianqiliu2000@163.com (T.L.); yuna009862@163.com (N.Y.); 18945631101@163.com (R.Z.); luozp0829@163.com (Z.L.); 2 Department of Gastroenterology, The First Affiliated Hospital of China Medical University, Shenyang 110001, China; 3 Teaching Center for Medical Experiment, China Medical University, Shenyang 110001, China

**Keywords:** MBNL1, intestinal fibrosis, RAS-MAPK pathway, Crohn’s disease

## Abstract

**Background:** Intestinal fibrosis is a severe complication of Crohn’s disease (CD) with no effective therapies currently available. Muscleblind-like protein 1 (MBNL1) is an RNA-binding protein that has been implicated in fibrosis across multiple organs, but its role in CD-associated intestinal fibrosis remains unexplored. This study aims to investigate the expression, functional role, and underlying mechanism of MBNL1 in intestinal fibrosis. **Methods:** MBNL1 expression was examined in a TNBS-induced mouse model and in stenotic intestinal tissues from CD patients. In vitro, human colonic fibroblasts (CCD-18Co) were stimulated with transforming growth factor-β1 (TGF-β1) to model fibrosis. MBNL1 was knocked down or overexpressed to assess its effects on fibroblast activation, proliferation (5-ethynyl-2′-deoxyuridine, EdU; Cell Counting Kit-8, CCK-8), and apoptosis (flow cytometry). Potential downstream pathways were predicted using BioGRID and DAVID analyses and validated by Western blot. A rescue experiment with the RAS activator ML-097 was performed to confirm pathway dependency. **Results:** MBNL1 expression was significantly upregulated in fibrotic tissues from both the mouse model and CD patients, as well as in TGF-β1-stimulated CCD-18Co. MBNL1 knockdown suppressed TGF-β1-induced fibroblast activation and proliferation while promoting apoptosis, whereas MBNL1 overexpression had the opposite effect. Mechanistically, MBNL1 positively regulated the RAS-MAPK signaling pathway. Reactivation of this pathway with ML-097 reversed the inhibitory effects of MBNL1 knockdown on fibroblast activation and proliferation. **Conclusions:** MBNL1 promotes colonic fibroblast activation and proliferation by activating the RAS-MAPK signaling pathway, establishing it as a potential therapeutic target for intestinal fibrosis in Crohn’s disease.

## 1. Introduction

Intestinal fibrosis is a serious complication of Crohn’s disease (CD). Its main pathological features include excessive deposition of extracellular matrix (ECM), thickening of the intestinal wall, and narrowing of the lumen, which can lead to severe complications, including intestinal obstruction and perforation. These complications are the primary reason patients often require repeated surgeries, which significantly impact their prognosis and quality of life [1,2]. Currently, there are no drugs available in clinical practice that can effectively reverse or slow the progression of fibrosis. The main treatment is surgical resection of the affected intestinal segment, but this approach is associated with high recurrence rates and numerous adverse effects [3]. Therefore, understanding the mechanisms underlying intestinal fibrosis and identifying potential molecular targets are essential for developing more effective therapies. The main pathological process involves continuous stimulation of the intestine by inflammatory or damaging signals, leading to abnormal activation of lamina propria fibroblasts [4]. These fibroblasts proliferate excessively, resist apoptosis, and differentiate into myofibroblasts. These cells produce excessive amounts of fibrosis-related proteins, such as alpha-smooth muscle actin (α-SMA), and secrete large quantities of ECM components, including types I and III collagen. This ultimately causes structural abnormalities and impairs intestinal function [5]. Current research primarily focuses on how signaling pathways, such as transforming growth factor-β (TGF-β) and Wnt, regulate gene transcription. However, growing evidence suggests that post-transcriptional regulation plays a more critical role in determining cell fate and adapting to environmental changes [6,7].

Muscleblind-like protein 1 (MBNL1) is a highly conserved RNA-binding protein and a member of the MBNL protein family. Its primary functions are to regulate alternative splicing and mRNA stability [8]. Recently, MBNL1’s role in fibrotic diseases has received more attention. Studies have shown that MBNL1 is involved in cardiac remodeling and can bind to and regulate transcription factors, such as serum response factor (SRF) and calcineurin Aβ, which promote myofibroblast differentiation [9]. Disrupting the MBNL1 binding site within the 3′ untranslated region (3′UTR) of the *SRF* gene using gene editing inhibits myofibroblast differentiation. Deleting the *Srf* gene in fibroblasts impairs wound healing and fibrosis. Furthermore, Bugg et al. identified MBNL1 as a key regulator of cardiac and skin fibrosis and elucidated its mechanism. They demonstrated that MBNL1 promotes fibrotic responses by binding to and regulating the mRNA of genes related to the cytoskeleton and ECM; MBNL1 knockdown reduces fibroblast activation and fibrosis [10]. Gene Set Enrichment Analysis (GSEA) has revealed an association between aberrant MBNL1 expression and the prognosis of idiopathic pulmonary fibrosis [11]. In renal fibrosis under high-glucose conditions, MBNL1 participates in the *Circ_Arf3/miR-452-5p/Mbnl1* axis, influencing the proliferation and expression of fibrosis-related proteins in mouse mesangial cells [12]. Notably, MBNL1 has also been associated with infantile hypertrophic pyloric stenosis, a condition characterized by muscle hypertrophy and fibrosis [13]. These findings suggest that MBNL1 plays an important role in fibrosis across various organs. However, its specific expression pattern, biological function, and molecular mechanism in CD-associated intestinal fibrosis remain unclear. One clinical study has provided initial clues indicating a connection between the methylation status of the *MBNL1* gene in the peripheral blood leukocytes of patients with CD and postoperative recurrence [14]. Recent transcriptomic and proteomic studies have identified novel signatures in fibrostenotic Crohn’s disease [2,15,16]. None of these studies highlighted MBNL1 as a top candidate. One possible reason is that those studies used bulk tissue or different cell populations, whereas we focused specifically on colonic fibroblasts. Our work provides experimental evidence that MBNL1 acts via the RAS-MAPK pathway in these cells, complementing the existing omics datasets.

This study aimed to investigate the expression, function, and molecular mechanism of MBNL1 in intestinal fibrosis using human clinical samples, animal models, and in vitro cellular experiments. The goal was to identify potential therapeutic targets for clinical intervention.

## 2. Materials and Methods

### 2.1. Human Intestinal Tissue

This study was approved by the Medical Science Research Ethics Committee of the First Affiliated Hospital of China Medical University (Approval No. [2024]2024-772-2). All participants provided written informed consent before sample collection. Intestinal tissues were obtained from 12 patients with CD-associated intestinal fibrosis. For each patient, paired tissue samples were collected under endoscopy from the stenotic region and the adjacent non-stenotic region, which was free of visible inflammation or ulceration. Tissues were either fixed in 4% paraformaldehyde for histological analysis or immediately snap-frozen in liquid nitrogen and stored at −80 °C for subsequent RNA and protein extraction. The clinical characteristics of the 12 CD patients are summarized in Table S1.

### 2.2. Mouse Model of Intestinal Fibrosis

All animal experiments were approved by the Experimental Animal Welfare and Ethics Committee of China Medical University (Approval No. CMU20240491). Eight-week-old female ICR mice were randomly divided into three groups (*n* = 6 per group): normal control (NC), ethanol vehicle control (Ethanol), and TNBS model (TNBS). The TNBS group was sensitized by epicutaneous application of 1% TNBS to the shaved dorsal skin, followed by weekly intracolonic administration of TNBS (0.75–2.5%, wt/vol) in 50% ethanol for six consecutive weeks. The Ethanol group received 50% ethanol alone, while the NC group received sterile saline. Disease Activity Index (DAI) scores were assessed based on weight loss, stool consistency, and fecal occult blood. Fecal occult blood was detected using the benzidine semi-quantitative test kit (Baso, Zhuhai, China). Briefly, a small amount of fresh feces was collected with a sterile sampler and evenly smeared onto the sample area of the test card. Developer A and developer B were then added sequentially. Timing was started immediately after adding developer B, and the result was read within 2 min. The color reaction was graded as follows: negative (−): no change within 2 min; positive (+): purplish red within 1–2 min; positive (++): purplish red within 1 min; positive (+++): bluish purple within 10 s; positive (++++): bluish purple immediately. The DAI scoring criteria are summarized in Table S2 [17]. All 6 mice in the TNBS group developed histologically confirmed intestinal fibrosis. Colon tissues were collected for histological analysis and molecular assays.

### 2.3. Cell Culture and Treatment

Human normal colonic fibroblasts (CCD-18Co, BFB) were cultured in DMEM supplemented with 10% fetal bovine serum and 1% penicillin-streptomycin at 37 °C in 5% CO_2_. Cells were authenticated by short tandem repeat (STR) profiling.

To establish an in vitro fibrosis model, cells were treated with 10 ng/mL transforming growth factor-β1 (TGF-β1) for 48 h based on preliminary optimization experiments. For MBNL1 knockdown, cells were transfected with specific siRNA targeting MBNL1 (siRNA-MBNL1-1831) or negative control siRNA (siRNA-NC) using Lipo6000 transfection reagent (Beyotime, Shanghai, China). For MBNL1 overexpression, cells were transfected with a pcDNA3.1-based MBNL1 overexpression plasmid or empty vector (Hai Xing Biotech, Suzhou, China). Transfections were performed according to the manufacturer’s instructions, and cells were harvested 48–72 h post-transfection for subsequent experiments. For CCK-8 assays, cells were seeded at 5 × 10^3^ per well in 96-well plates. For Western blot and EdU assays, cells were seeded at 2 × 10^5^ per well in 6-well plates.

### 2.4. Histopathological Analysis

Mouse colon and human intestinal tissues were fixed in 4% paraformaldehyde, embedded in paraffin, and sectioned at 4 μm thickness.

For H&E staining, sections were stained with hematoxylin and eosin and examined under a light microscope. Histological damage in mouse colon tissues was scored according to previously established criteria based on the extent of injury, lesion depth, crypt destruction, and inflammation severity (Table S3) [18].

For Masson’s trichrome staining, sections were stained using a commercial kit (Solarbio, Beijing, China). Collagen deposition was quantified as the percentage of positive area using ImageJ software (version 1.53g, National Institutes of Health, Bethesda, MD, USA).

For immunohistochemistry (IHC), sections were subjected to antigen retrieval, blocked, and incubated with primary antibodies against α-SMA (Proteintech, Wuhan, China) and MBNL1 (Abways, Shanghai, China), followed by HRP-conjugated secondary antibodies. Staining was visualized with DAB and counterstained with hematoxylin. Positive staining area was quantified using ImageJ software.

### 2.5. Transcriptome Sequencing and Bioinformatics Analysis

Total RNA was extracted from colon tissues of control and TNBS-treated mice (*n* = 3 per group) using TRIzol reagent (Thermo Fisher Scientific, Waltham, MA, USA). RNA quality was assessed, and qualified samples were used to construct cDNA libraries, which were sequenced on an Illumina platform (Illumina, San Diego, CA, USA). Raw reads were filtered and aligned to the mouse reference genome. Differentially expressed genes (DEGs) were identified using criteria of |log_2_(fold change)| > 1 and adjusted *p* < 0.05. Gene Ontology (GO) and Kyoto Encyclopedia of Genes and Genomes (KEGG) pathway enrichment analyses were performed to assess functional and pathway annotations.

### 2.6. RNA Extraction and Real-Time Quantitative PCR (RT-qPCR)

Total RNA was extracted from mouse colon tissues and cultured CCD-18Co using TRIzol reagent (Vazyme, Nanjing, China). Total RNA (1 μg) was reverse-transcribed into cDNA in a 20 μL reaction volume using the HiScript II Q RT SuperMix for qPCR (Vazyme, Nanjing, China). RT-qPCR was conducted using ChamQ Universal SYBR qPCR Master Mix (Vazyme, Nanjing, China) on a real-time PCR system. The thermal cycling protocol was: 95 °C for 30 s, followed by 40 cycles of 95 °C for 10 s and 60 °C for 30 s. Melting curve analysis confirmed amplification specificity. Gene expression levels were normalized to GAPDH, and relative expression was calculated using the 2^−ΔΔCt^ method. All reactions were performed in triplicate. The primer sequences are listed in Table S4.

### 2.7. Western Blot

Protein samples were extracted from mouse colon tissues and cultured CCD-18Co using RIPA lysis buffer (Beyotime, Shanghai, China) containing protease and phosphatase inhibitors. Protein concentrations were determined using a BCA kit (Abbkine, Wuhan, China). SDS-PAGE separated equal amounts of protein (15 μg), transferred onto PVDF membranes (Millipore, Burlington, MA, USA), and blocked with 5% non-fat milk or 5% BSA in TBST. Membranes were incubated overnight at 4 °C with primary antibodies, followed by HRP-conjugated secondary antibodies for 1 h. Protein bands were visualized using an ECL kit (Tanon, Shanghai, China). Band intensities were quantified using ImageJ software and normalized to GAPDH.

The following primary antibodies were used: rabbit anti-MBNL1 (1:1500, Abways, Shanghai, China, CY8483), rabbit anti-FN1 (1:10000, Proteintech, Wuhan, China, 15613-1-AP), rabbit anti-COL1A1 (1:1000, Abways, Shanghai, China, CY5120), rabbit anti-α-SMA (1:8000, Proteintech, Wuhan, China, 144395-1-AP), rabbit anti-RAS (1:1000, Abmart, Shanghai, China, T56672), rabbit anti-RAF (1:1500, Abways, Shanghai, China, CY5225), rabbit anti-p-RAF (S259) (1:1500, Abways, Shanghai, China, CY5944), rabbit anti-MEK1 (1:1500, Abways, Shanghai, China, CY5854), rabbit anti-p-MEK1 (S298) (1:1500, Abways, Shanghai, China, CY5042), rabbit anti-ERK1/2 (1:3000, Bioss, Beijing, China, bsm-52259R), rabbit anti-p-ERK1/2 (Thr202/Tyr204) (1:1000, Bioss, Beijing, China, bsm-63322R), rabbit anti-BAX (1:1500, Abways, Shanghai, China, CY5059), rabbit anti-BCL2 (1:1000, Abmart, Shanghai, China, T40056), and rabbit anti-GAPDH (1:10000, Abclonal, Wuhan, China, A19056).

### 2.8. Bioinformatic Prediction of MBNL1 Targets

To identify potential downstream targets and pathways regulated by MBNL1, protein-protein interaction (PPI) data were retrieved from the BioGRID database (https://thebiogrid.org (accessed on 9 April 2026)). The identified interacting proteins were subjected to Gene Ontology (GO) enrichment and Kyoto Encyclopedia of Genes and Genomes (KEGG) pathway enrichment analyses using the DAVID bioinformatics resource (https://david.ncifcrf.gov (accessed on 9 April 2026)). Enriched terms with a *p* value < 0.05 were considered statistically significant.

### 2.9. CCK-8 Assay

CCD-18Co were seeded into 96-well plates at a density of 5 × 10^3^ cells per well and cultured overnight. After the indicated treatments, 10 μL of CCK-8 solution was added to each well and incubated for 2 h at 37 °C. Absorbance was measured at 450 nm using a microplate reader (Abbkine, Wuhan, China). Each experiment was performed in triplicate.

### 2.10. EdU Assay

CCD-18Co were seeded into 6-well plates. After the indicated treatments, cells were incubated with 5 μM EdU for 2 h, then fixed, permeabilized, and stained using an EdU-488 kit (Meilunbio, Dalian, China) according to the manufacturer’s protocol. Nuclei were counterstained with Hoechst 33342. For each experimental condition, at least five random fields were captured at 20× objective (scale bar = 50 μm) using a fluorescence microscope (Nikon, Tokyo, Japan). The number of EdU-positive cells and Hoechst-positive nuclei was counted in each field, and the percentage of EdU-positive cells was calculated. Each experiment was performed independently three times.

### 2.11. Statistical Analysis

All quantitative data are presented as the mean ± standard deviation (SD). Each experiment was independently repeated at least three times, with three technical replicates per experiment. Statistical analyses were performed using GraphPad Prism version 10 (GraphPad Software, Boston, MA, USA). Comparisons between two groups were analyzed using independent samples *t*-test. Comparisons among multiple groups were analyzed using one-way analysis of variance (ANOVA), followed by Tukey’s post hoc test for pairwise comparisons with adjustment for multiple testing. When data did not meet the assumptions of normality or homogeneity of variance, the Kruskal–Wallis test with Dunn’s post hoc test was applied. A *p* value < 0.05 was considered statistically significant.

## 3. Results

### 3.1. MBNL1 Is Upregulated in the TNBS-Induced Mouse Model of Intestinal Fibrosis

**Figure 1:**
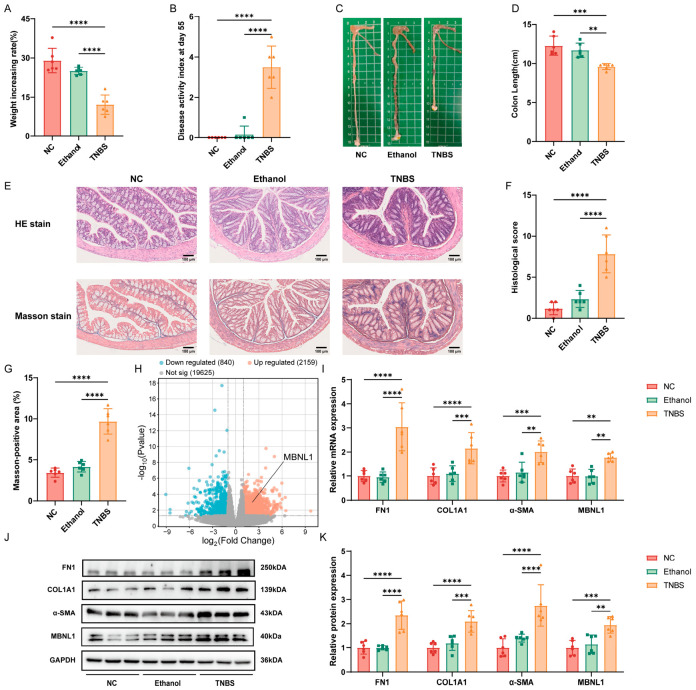
MBNL1 is upregulated in the TNBS-induced mouse model of intestinal fibrosis. (**A**) Body weight gain rate. (**B**) DAI scores. (**C**) Representative images of colon gross morphology. (**D**) Colon length. (**E**) Representative images of H&E and Masson staining of colon tissues (scale bar = 100 μm). (**F**) Histopathological scores. (**G**) Collagen-positive area ratio. (**H**) Volcano plot of differentially expressed genes from transcriptome sequencing; MBNL1 is highlighted as a significantly upregulated gene. (**I**) Relative mRNA expression levels of FN1, COL1A1, α-SMA, and MBNL1. (**J**) Representative Western blot bands of FN1, COL1A1, α-SMA, and MBNL1. (**K**) Relative protein expression levels of FN1, COL1A1, α-SMA, and MBNL1. Data are presented as the mean ± SD (*n* = 6). Comparisons among multiple groups were performed using one-way ANOVA followed by Tukey’s post hoc test. ** *p* < 0.01; *** *p* < 0.001; **** *p* < 0.0001.

To investigate the molecular mechanisms of intestinal fibrosis, a TNBS-induced mouse model was established. TNBS-treated mice exhibited lower body weight gain, higher DAI scores, shorter colon length, and increased inflammatory infiltration, crypt destruction, and collagen deposition compared with controls (Figure 1A–G). Consistently, the expression of fibrosis markers FN1, COL1A1, and α-SMA was significantly upregulated at both mRNA and protein levels (Figure 1I–K), confirming successful model establishment.

Transcriptome sequencing was performed to identify potential molecular targets. Analysis of colon tissues from control and TNBS-treated mice revealed distinct gene expression profiles, with differentially expressed genes enriched in fibrosis-related pathways, including calcium, cAMP, and cGMP-PKG signaling (Figure S1). Among the upregulated genes, the RNA-binding protein MBNL1 was identified as a candidate (Figure 1H). RT-qPCR and Western blot validation confirmed that MBNL1 expression was significantly elevated in fibrotic colon tissues (Figure 1I–K), suggesting its involvement in intestinal fibrosis.

### 3.2. MBNL1 Is Specifically Upregulated in Stenotic Intestinal Tissues from Crohn’s Disease Patients

**Figure 2:**
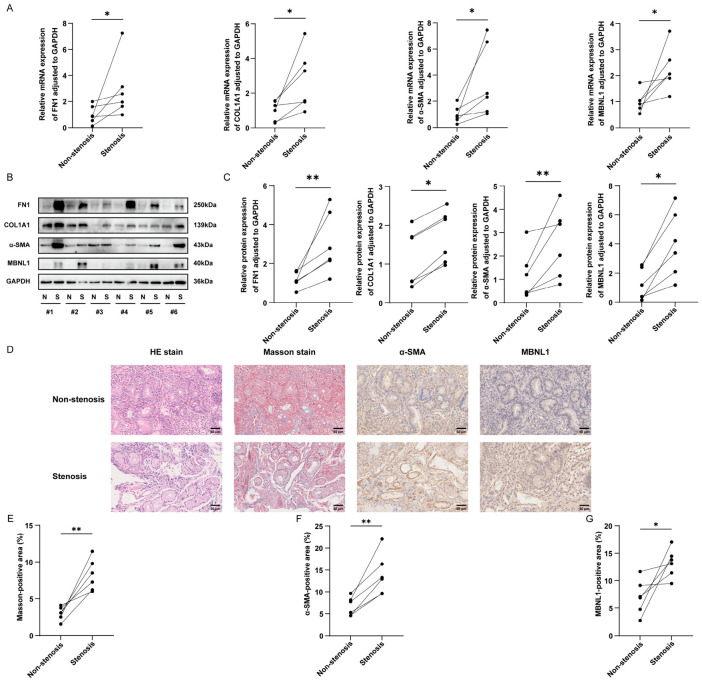
MBNL1 is specifically upregulated in stenotic intestinal tissues from CD patients. (**A**) Relative mRNA expression levels of FN1, COL1A1, α-SMA, and MBNL1. (**B**) Representative Western blot bands of FN1, COL1A1, α-SMA, and MBNL1. (**C**) Relative protein expression levels of FN1, COL1A1, α-SMA, and MBNL1. (**D**) Representative images of H&E staining, Masson staining, α-SMA immunohistochemistry, and MBNL1 immunohistochemistry (scale bar = 50 μm). (**E**) Collagen-positive area ratio. (**F**) α-SMA-positive area ratio. (**G**) MBNL1-positive area ratio. Data are presented as the mean ± SD (paired samples, *n* = 6). Comparisons between two groups were performed using paired *t*-tests. * *p* < 0.05; ** *p* < 0.01.

To assess the clinical relevance of MBNL1 in CD-associated fibrosis, paired stenotic and non-stenotic intestinal tissues from 12 CD patients were analyzed. RT-qPCR and Western blot revealed that MBNL1 mRNA and protein levels were significantly elevated in stenotic tissues compared with non-stenotic tissues, concurrent with increased expression of fibrosis markers FN1, COL1A1, and α-SMA (Figure 2A–C).

Histological examination was performed to characterize tissue alterations. H&E staining showed marked structural disruption and extensive inflammatory infiltration in the mucosa and submucosa of stenotic regions (Figure 2D). Masson staining revealed increased collagen deposition, with a significantly higher fibrotic area ratio in stenotic tissues (Figure 2D,E). Immunohistochemistry demonstrated enhanced α-SMA expression in myofibroblasts and stronger MBNL1 staining, predominantly nuclear and cytoplasmic, in stenotic regions (Figure 2D,F,G). Collectively, these results indicate that MBNL1 is specifically upregulated in fibrotic tissues from CD patients and is closely associated with intestinal fibrosis progression.

### 3.3. TGF-β1 Induces Fibrosis and Upregulates MBNL1 Expression in Colonic Fibroblasts

**Figure 3:**
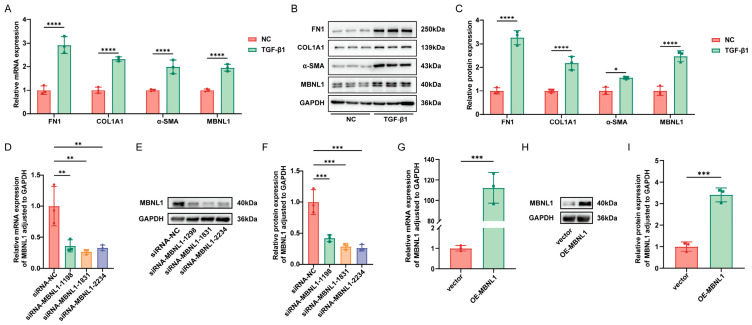
TGF-β1 induces fibrosis and upregulates MBNL1 expression in colonic fibroblasts, with validation of MBNL1 knockdown and overexpression efficiency. (**A**) Relative mRNA expression levels of MBNL1, FN1, COL1A1, and α-SMA after TGF-β1 stimulation. (**B**) Representative Western blot bands of MBNL1, FN1, COL1A1, and α-SMA. (**C**) Relative protein expression levels of MBNL1, FN1, COL1A1, and α-SMA. (**D**) MBNL1 mRNA expression levels after transfection with siRNA-MBNL1. (**E**) Representative MBNL1 protein bands after transfection with siRNA-MBNL1. (**F**) MBNL1 protein expression levels after transfection with siRNA-MBNL1. (**G**) MBNL1 mRNA expression levels after transfection with MBNL1 overexpression plasmid. (**H**) Representative MBNL1 protein bands after transfection with MBNL1 overexpression plasmid. (**I**) MBNL1 protein expression levels after transfection with MBNL1 overexpression plasmid. Data are presented as the mean ± SD (*n* = 3). Comparisons between two groups were performed using independent-samples *t*-test; multiple-group comparisons were performed using one-way ANOVA with Tukey’s post hoc test. * *p* < 0.05; ** *p* < 0.01; *** *p* < 0.001; **** *p* < 0.0001.

To establish an in vitro model of intestinal fibrosis, CCD-18Co were treated with 10 ng/mL TGF-β1 for 48 h. Compared with untreated controls, TGF-β1 stimulation significantly upregulated the mRNA and protein levels of fibrosis markers FN1, COL1A1, and α-SMA (Figure 3A–C), confirming successful model establishment. Notably, MBNL1 expression was also markedly increased at both mRNA and protein levels (Figure 3A–C), consistent with the in vivo observations.

To investigate the functional role of MBNL1, loss- and gain-of-function cell models were generated. Among three siRNAs targeting MBNL1, siRNA-MBNL1-1831 exhibited the highest knockdown efficiency and was selected for subsequent experiments (Figure 3D–F). Transfection with an MBNL1 overexpression plasmid resulted in significantly elevated MBNL1 mRNA and protein levels compared with empty vector controls (Figure 3G–I).

### 3.4. MBNL1 Promotes Activation and Proliferation of Colonic Fibroblasts

To investigate the functional role of MBNL1 in fibroblast biology, loss- and gain-of-function experiments were performed under TGF-β1 stimulation.

**Figure 4:**
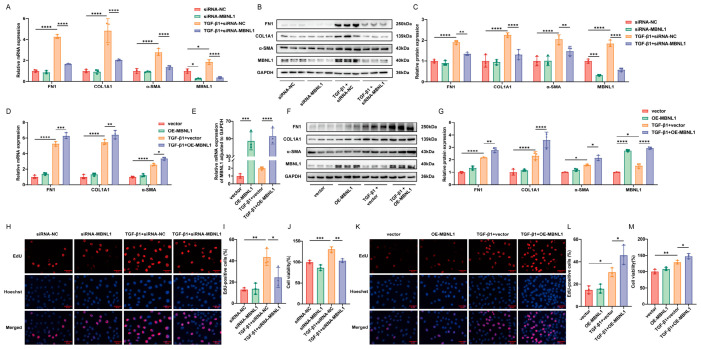
MBNL1 promotes activation and proliferation of colonic fibroblasts. (**A**–**C**) Effect of MBNL1 knockdown on fibroblast activation. (**A**) Relative mRNA expression levels of FN1, COL1A1, α-SMA, and MBNL1. (**B**) Representative Western blot bands. (**C**) Relative protein expression levels. (**D**–**G**) Effect of MBNL1 overexpression on fibroblast activation. (**D**) Relative mRNA expression levels of FN1, COL1A1, and α-SMA. (**E**) Relative MBNL1 mRNA expression levels. (**F**) Representative Western blot bands. (**G**) Relative protein expression levels. (**H**–**J**) Effect of MBNL1 knockdown on fibroblast proliferation. (**H**) Representative EdU staining images (scale bar = 50 μm). (**I**) EdU-positive cell ratio. (**J**) Cell viability measured by CCK-8 assay. (**K**–**M**) Effect of MBNL1 overexpression on fibroblast proliferation. (**K**) Representative EdU staining images (scale bar = 50 μm). (**L**) EdU-positive cell ratio. (**M**) Cell viability measured by CCK-8 assay. Data are presented as the mean ± SD (*n* = 3). Comparisons among multiple groups were performed using one-way ANOVA followed by Tukey’s post hoc test. * *p* < 0.05; ** *p* < 0.01; *** *p* < 0.001; **** *p* < 0.0001.

Knockdown of MBNL1 significantly suppressed TGF-β1-induced fibroblast activation. Compared with the TGF-β1 + siRNA-NC group, the TGF-β1 + siRNA-MBNL1 group showed reduced mRNA and protein levels of fibrosis markers FN1, COL1A1, and α-SMA (Figure 4A–C). Conversely, MBNL1 overexpression enhanced activation, with further upregulation of these markers in the TGF-β1 + OE-MBNL1 group compared with the TGF-β1 + vector group (Figure 4D–G).

Proliferation assays revealed that MBNL1 knockdown inhibited TGF-β1-induced cell proliferation, as evidenced by decreased EdU-positive cell ratios and reduced cell viability (Figure 4H–J). In contrast, MBNL1 overexpression promoted proliferation, as shown by increased EdU-positive cell ratios and cell viability (Figure 4K–M).

Apoptosis analysis by flow cytometry showed that MBNL1 knockdown increased the apoptosis rate, accompanied by upregulation of the pro-apoptotic protein BAX, downregulation of the anti-apoptotic protein BCL-2, and a reduced BCL-2/BAX ratio (Figure S2). Conversely, MBNL1 overexpression decreased apoptosis and increased the BCL-2/BAX ratio (Figure S3).

Collectively, these gain- and loss-of-function experiments demonstrate that MBNL1 promotes fibroblast activation and proliferation while inhibiting apoptosis, establishing it as a key regulator in intestinal fibrosis progression.

### 3.5. MBNL1 Positively Regulates the RAS-MAPK Signaling Pathway

**Figure 5:**
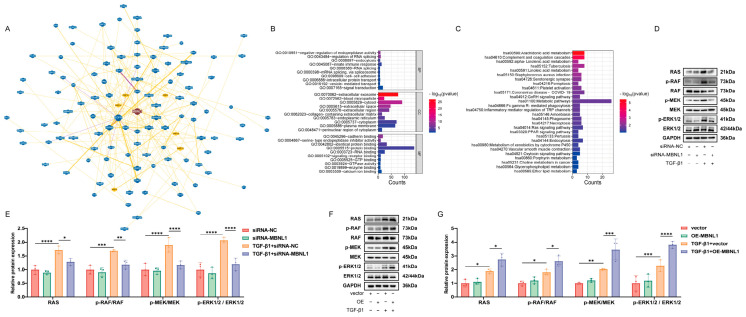
MBNL1 positively regulates the RAS-MAPK signaling pathway. (**A**) Protein-protein interaction (PPI) network of MBNL1 generated from the BioGRID database. (**B**) GO functional enrichment analysis of potential MBNL1 downstream targets using DAVID. (**C**) KEGG pathway enrichment analysis of potential MBNL1 downstream targets showing enrichment in the RAS signaling pathway. (**D**) Representative Western blot bands of RAS, RAF, p-RAF, MEK, p-MEK, ERK1/2, and p-ERK1/2 in CCD-18Co after MBNL1 knockdown. (**E**) Relative protein expression levels of RAS, RAF, p-RAF, MEK, p-MEK, ERK1/2, and p-ERK1/2 after MBNL1 knockdown. (**F**) Representative Western blot bands of RAS, RAF, p-RAF, MEK, p-MEK, ERK1/2, and p-ERK1/2 in CCD-18Co after MBNL1 overexpression. (**G**) Relative protein expression levels of RAS, RAF, p-RAF, MEK, p-MEK, ERK1/2, and p-ERK1/2 after MBNL1 overexpression. Data are presented as the mean ± SD (*n* = 3). Comparisons among groups were performed using one-way ANOVA followed by Tukey’s post hoc test. * *p* < 0.05; ** *p* < 0.01; *** *p* < 0.001; **** *p* < 0.0001.

To elucidate the molecular mechanism by which MBNL1 promotes intestinal fibrosis, potential downstream targets were predicted using the BioGRID database. Protein-protein interaction (PPI) network analysis suggested that MBNL1 may interact with proteins involved in RNA metabolism, splicing regulation, and signal transduction (Figure 5A). GO analysis revealed enrichment in RNA splicing, cell junctions, and signal transduction (Figure 5B), consistent with its role as an RNA-binding protein. KEGG pathway enrichment further identified significant enrichment of potential MBNL1 targets in the RAS signaling pathway (Figure 5C).

Based on these predictions, the effect of MBNL1 on RAS-MAPK pathway activity was examined. Western blot analysis showed that MBNL1 knockdown significantly reduced the levels of RAS, p-RAF, p-MEK, and p-ERK1/2, while total RAF, MEK, and ERK1/2 remained unchanged. Consequently, the p-RAF/RAF, p-MEK/MEK, and p-ERK1/2/ERK1/2 ratios were markedly decreased (Figure 5D,E). Conversely, MBNL1 overexpression significantly increased the phosphorylation levels of these proteins (Figure 5F,G). These findings indicate that MBNL1 acts as an upstream positive regulator of the RAS-MAPK signaling pathway, suggesting that it may exert its pro-fibrotic effects by activating this pathway.

### 3.6. RAS Pathway Activation Rescues MBNL1 Knockdown-Induced Defects in Fibroblast Activation and Proliferation

**Figure 6:**
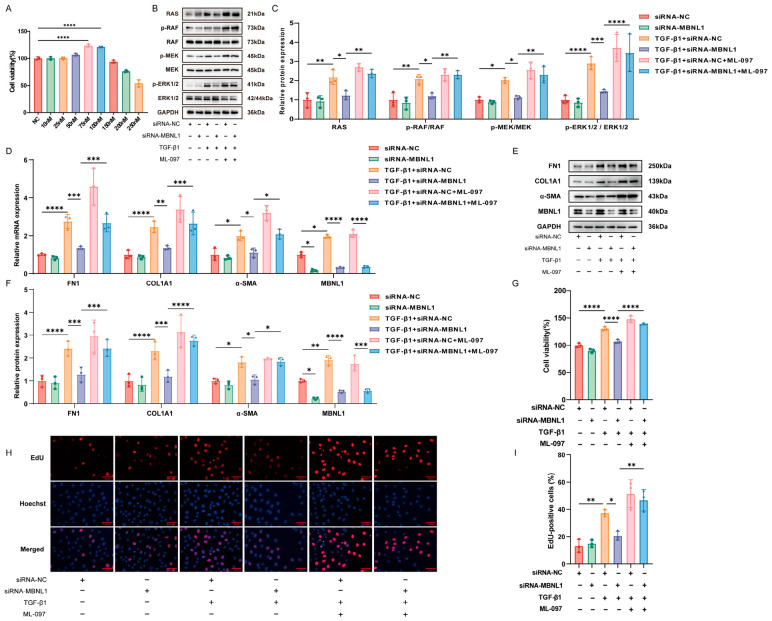
RAS pathway activation rescues defects in fibroblast activation and proliferation induced by MBNL1 knockdown. (**A**) Optimal concentration of ML-097 determined by CCK-8 assay. (**B**) Representative Western blot bands of RAS, RAF, p-RAF, MEK, p-MEK, ERK1/2, and p-ERK1/2 in MBNL1-knockdown CCD-18Co after ML-097 treatment. (**C**) Relative protein expression levels of RAS, RAF, p-RAF, MEK, p-MEK, ERK1/2, and p-ERK1/2 in MBNL1-knockdown CCD-18Co after ML-097 treatment. (**D**) Relative mRNA expression levels of FN1, COL1A1, α-SMA, and MBNL1 in MBNL1-knockdown CCD-18Co after ML-097 treatment. (**E**) Representative Western blot bands of FN1, COL1A1, α-SMA, and MBNL1 in MBNL1-knockdown CCD-18Co after ML-097 treatment. (**F**) Relative protein expression levels of FN1, COL1A1, α-SMA, and MBNL1 in MBNL1-knockdown CCD-18Co after ML-097 treatment. (**G**) Cell viability measured by CCK-8 assay in MBNL1-knockdown CCD-18Co after ML-097 treatment. (**H**) Representative EdU staining images in MBNL1-knockdown CCD-18Co after ML-097 treatment (scale bar = 50 μm). (**I**) EdU-positive cell ratio in MBNL1-knockdown CCD-18Co after ML-097 treatment. Data are presented as the mean ± SD (*n* = 3). Comparisons among groups were performed using one-way ANOVA followed by Tukey’s post hoc test. * *p* < 0.05; ** *p* < 0.01; *** *p* < 0.001; **** *p* < 0.0001.

To determine whether the RAS-MAPK pathway is required for MBNL1-mediated fibroblast activation and proliferation, rescue experiments were performed using the RAS activator ML-097. The optimal concentration of ML-097 was determined to be 75 nM (Figure 6A). Western blot analysis confirmed that ML-097 treatment significantly restored the reduced phosphorylation levels of RAF, MEK, and ERK1/2 induced by MBNL1 knockdown (Figure 6B,C). Functional rescue experiments demonstrated that ML-097 markedly rescued the downregulation of fibrosis markers FN1, COL1A1, and α-SMA (Figure 6D–F) and the suppression of cell proliferation (Figure 6G–I) caused by MBNL1 knockdown. In contrast, ML-097 did not significantly rescue the increased apoptosis induced by MBNL1 knockdown, suggesting that while the RAS-MAPK pathway is essential for MBNL1-regulated activation and proliferation, MBNL1 may regulate apoptosis through distinct mechanisms.

**Figure 7:**
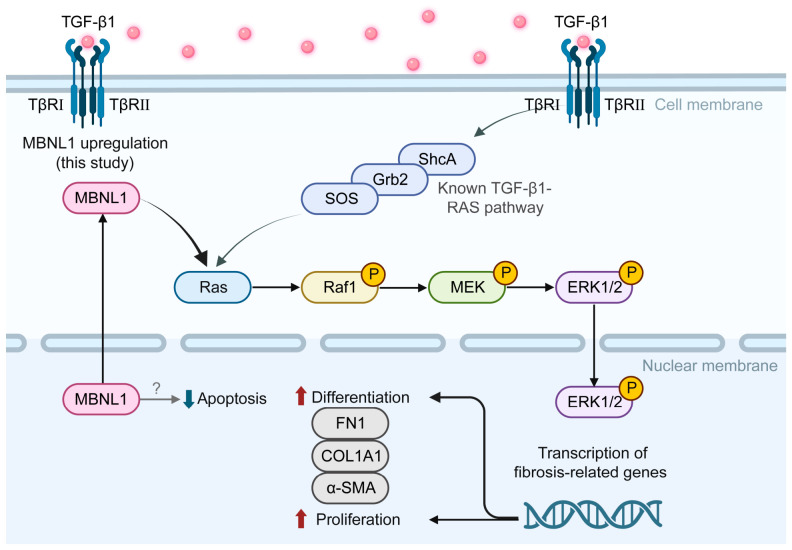
Schematic model of MBNL1-mediated intestinal fibrosis. Upon TGF-β1 stimulation, the ligand binds to TβRII and TβRI, initiating the classical ShcA/Grb2/SOS cascade, which leads to RAS activation (known TGF-β1-RAS pathway). In parallel, TGF-β1 upregulates MBNL1 expression. MBNL1 then positively regulates the RAS-MAPK signaling pathway (RAS-RAF-MEK-ERK), thereby promoting fibroblast activation and proliferation. This study identifies MBNL1 as a key positive regulator of the RAS-MAPK pathway in intestinal fibrosis. MBNL1 also regulates apoptosis through a RAS-MAPK-independent mechanism. Created in BioRender. Zhang, L. (2026) https://BioRender.com/emdj16e, accessed on 9 April 2026.

Based on these findings, a schematic model illustrating the mechanism by which MBNL1 promotes intestinal fibrosis is proposed (Figure 7).

## 4. Discussion

This study integrates clinical samples, animal models, and in vitro experiments to identify MBNL1 as a critical regulator of CD-associated intestinal fibrosis. Mechanistically, MBNL1 promotes colonic fibroblast activation and proliferation by positively regulating the RAS-MAPK signaling pathway, while its regulation of apoptosis appears to operate independently. These findings extend the pro-fibrotic role of MBNL1 to the intestine and suggest a potential therapeutic target for fibrostenotic CD.

Functional studies revealed that MBNL1 promotes fibroblast activation and proliferation via the RAS-MAPK pathway. Knockdown of MBNL1 reduced phosphorylation of RAS, RAF, MEK, and ERK, whereas overexpression enhanced it. Importantly, rescue experiments with the RAS activator ML-097 reversed the effects of MBNL1 knockdown, suggesting that MBNL1 acts upstream of this pathway [19].TGF-β1, a master regulator of fibrosis, activates both canonical Smad and non-canonical pathways, including RAS-MAPK [20,21]. We propose that TGF-β1 activates RAS-MAPK in a rapid but transient manner, while simultaneously upregulating MBNL1, which sustains and amplifies pathway activity. We hypothesize that MBNL1 may bind to and stabilize mRNAs encoding key RAS-MAPK components, such as RAS guanine nucleotide exchange factors, thereby maintaining prolonged signaling [22,23]. Alternatively, it may influence the generation of splice variants or expression levels of certain GTPase-activating proteins, relieving RAS inhibition. We speculate that this mechanism could ensure that even as the initial stimulus wanes in a chronic inflammatory environment, fibroblast activation and proliferation persist, a critical feature for fibrosis progression [24]. These hypotheses warrant testing in future studies using RIP-seq to identify MBNL1-bound mRNAs.

An independent role for MBNL1 in regulating apoptosis was also identified. MBNL1 knockdown increased apoptosis and reduced the BCL-2/BAX ratio, whereas overexpression had the opposite effect. However, the RAS activator ML-097 failed to rescue these apoptotic phenotypes, indicating that MBNL1 regulates apoptosis through mechanisms distinct from the RAS-MAPK pathway. Given its function as an RNA-binding protein, MBNL1 may directly interact with mRNAs of apoptosis-related genes such as *BCL-X*, influencing alternative splicing or mRNA stability [25,26]. This hypothesis could be addressed by combining RIP-seq with RNA-seq to map MBNL1 targets involved in apoptosis. However, the precise mechanism by which MBNL1 regulates apoptosis remains unexplored and warrants further investigation.

From a therapeutic perspective, targeting MBNL1 offers a promising strategy for intestinal fibrosis. Current fibrosis treatments are increasingly shifting toward targeted therapies [16,27]. Compared with directly inhibiting broadly expressed kinases such as ERK, targeting an upstream RBP may provide a broader therapeutic window. However, RBP-targeted therapies face challenges related to tissue specificity, as MBNL1 plays essential roles in skeletal muscle and the central nervous system [28]. Systemic inhibition could lead to adverse effects, such as motor or cognitive impairments [29,30,31]. Nucleic acid-based approaches, such as antisense oligonucleotides or siRNA, represent a feasible direction [32,33,34]. RNAi drugs achieving liver-specific delivery through chemical modifications and delivery systems have been developed [35,36]. Developing similar technologies to target intestinal myofibroblasts will be critical for clinical translation, for example, using antibodies or ligands targeting specific markers on activated fibroblasts for drug conjugation [37,38,39].

Targeting MBNL1 could complement current anti-TNF or anti-integrin therapies by suppressing fibroblast activation, which is not achieved by most anti-inflammatory drugs. Unlike broad-spectrum kinase inhibitors, MBNL1 is an RNA-binding protein that could be modulated via antisense oligonucleotides or small interfering RNAs, offering a more specific approach with potentially fewer off-target effects [25]. However, a recent study showed that smooth muscle-specific knockout of *Mbnl1* leads to bowel dysmotility [40], suggesting that systemic inhibition of MBNL1 could cause gastrointestinal side effects. Therefore, targeted delivery of MBNL1 inhibitors to colonic fibroblasts, for example, using nanoparticles or antibody-drug conjugates, may be essential for future therapeutic development. Future studies should also explore whether MBNL1 inhibition synergizes with established anti-fibrotic agents such as pirfenidone or with anti-inflammatory drugs in preclinical models of intestinal fibrosis.

Several limitations should be acknowledged. In addition, the human tissue analysis was based on only 12 patient biopsies, which limits statistical power and generalizability. Future studies with larger cohorts are needed to confirm the clinical relevance of MBNL1. All in vitro experiments were conducted using the CCD-18Co cell line, which may not fully recapitulate the biology of primary human intestinal fibroblasts. Validation in primary cells isolated from patient tissues should be pursued in the future. Moreover, in vivo loss-of-function studies, for example, using fibroblast-specific Mbnl1 knockout or targeted siRNA delivery, are necessary to confirm the translational potential of MBNL1 inhibition. It should be noted that the mechanistic link between MBNL1 and the RAS-MAPK pathway, while supported by rescue experiments, remains indirect. We did not perform RNA-binding assays such as RIP-seq, nor did we conduct mRNA stability analyses, to identify direct targets of MBNL1. Therefore, our findings indicate a positive regulatory association rather than a proven upstream mechanism. Additionally, the direct mRNA targets of MBNL1 remain to be identified. While we have established that MBNL1 functions via the RAS-MAPK pathway, the specific mRNAs it binds and regulates within this pathway remain to be investigated, ideally using RIP-seq and transcriptomic integration. Future studies are required to determine whether MBNL1 directly binds to and stabilizes RAS pathway-related transcripts. Finally, patient heterogeneity in MBNL1 dependence should be explored, as not all patients with CD fibrosis may equally depend on MBNL1. Assessing MBNL1 activity, its specific splicing events, or downstream p-ERK levels in patient tissues could help identify candidates for targeted therapy, advancing precision medicine.

In summary, this study introduces MBNL1 as a positive regulator of intestinal fibrosis by activating the RAS-MAPK pathway and reveals its independent role in regulating apoptosis. These findings provide a solid foundation for developing targeted therapies aimed at reversing or halting fibrotic progression in CD.

## 5. Conclusions

This study demonstrates that MBNL1 expression is significantly upregulated in CD-associated intestinal fibrotic tissues and in TNBS-induced fibrotic mouse colon. Functional experiments reveal that MBNL1 promotes colonic fibroblast activation and proliferation while inhibiting apoptosis, thereby playing a critical role in the progression of intestinal fibrosis. Mechanistic studies indicate that MBNL1 mediates fibroblast activation and proliferation by positively regulating the RAS-MAPK signaling pathway, whereas its regulation of apoptosis likely involves distinct mechanisms. These findings identify MBNL1 as a potential therapeutic target for intestinal fibrosis.

## Data Availability

The datasets generated and analyzed during the current study are available from the corresponding author upon reasonable request.

## Supplementary Materials

The following supporting information can be downloaded at: https://www.mdpi.com/article/10.3390/biomedicines14061207/s1, Table S1: Clinical characteristics of the 12 Crohn’s disease patients with intestinal fibrosis; Table S2: DAI scoring criteria; Table S3: Histopathological scoring criteria for mouse colon tissue; Table S4: Primer sequences; Figure S1: Transcriptome sequencing and bioinformatics analysis of mouse colon tissue; Figure S2: Effect of MBNL1 knockdown on apoptosis in CCD-18Co; Figure S3: Effect of MBNL1 overexpression on apoptosis in CCD-18Co.

## Abbreviations

The following abbreviations are used in this manuscript:

BAX	BCL2-Associated X protein
BCL-2	B-cell lymphoma-2 gene
CD	Crohn’s disease
COL1A1	Collagen, type I, alpha 1
DAI	Disease activity index
DEGs	Difference Expression Genes
ECM	Extracellular Matrix
EdU	5-ethynyl-2′-deoxyuridine
ERK	Extracellular Regulated Protein Kinase
FN1	Fibronectin
GAPDH	Glyceraldehyde-3-Phosphate Dehydrogenase
HE	Hematoxylin and Eosin Staining
MAPK	Mitogen-Activated Protein Kinase
MBNL1	Muscleblind-like protein 1
MEK	Mitogen-activated protein
PCA	Principal components analysis
p-ERK	Phospho-Extracellular Regulated Protein Kinase
p-MEK	Phospho-mitogen-activated protein
p-RAF	Phospho- Serine/Threonine Kinase
RAS	Rat sarcoma
RAF	Serine/Threonine Kinase
TGF-β1	Transforming growth factor-β1
TNBS	2,4,6-Trinitrobenzenesulfonic acid solution
α-SMA	Alpha-smooth muscle actin
