# Supplementary material for: MBNL1 Promotes Intestinal Fibrosis via RAS-MAPK Pathway-Mediated Fibroblast Activation and Proliferation
Source: Biomedicines. 2026 May 27;14(6):1207. doi: 10.3390/biomedicines14061207 (PMC13297613; doi:10.3390/biomedicines14061207)
Supplement: Supplementary file 1 [file biomedicines-14-01207-s001.zip › biomedicines-4281871-supplementary.pdf]

Table S1. Clinical characteristics of the 12 Crohn's disease patients with intestinal fibrosis.

Characteristic	Category	n	%
**Age (years)**	18-45	4	33.3
	≥45	8	66.7
	Mean ± SD	48.2 ± 10.5	
**Sex**	Male	9	75.0
	Female	3	25.0
**CDAI score**[1]	<150	6	50.0
	≥150	6	50.0
	Median (IQR)[2]	145 (85–195)	
**Biopsy site**	Terminal ileum	9	75.0
	Colon	3	25.0
**Disease duration (years)**	Median (IQR)	4.5 (2.0–8.0)	
**Previous biologics use**	Yes	7	58.3
	No	5	41.7

[1] CDAI, Crohn's Disease Activity Index.[2] IQR, interquartile range.

Table S2. DAI scoring criteria.

Score	Weight Loss (%)	Stool Consistency	Occult Blood
0	None	Formed, hard	Negative (-)
1	1-5	Formed, soft	Positive (+)
2	6-10	Loose	Positive (++)
3	11-20	Mild diarrhea	Positive (+++)
4	>20	Severe diarrhea	Positive (++++)

Table S3. Histopathological scoring criteria for mouse colon tissue.

Score	Extent of Damage (%)	Depth of Lesion	Crypt Damage	Inflammation Severity
0	None	None	None	None
1	0-25	Mucosa	Basal 1/3 crypt damaged	Mild
2	25-50	Submucosa	Basal 2/3 crypt damaged	Moderate
3	50-75	Muscularis mucosa	Some epithelial cells intact	Severe
4	75-100	-	All crypts and epithelium destroyed	Very severe

Table S4. Primer sequences.

Gene Name	Forward	Reverse	Product Size (bp)
Mouse-GAPDH	CATCACTGCCACCCAGAAGACTG	ATGCCAGTGAGCTTCCCGTTCAG	153
Mouse-FN1	CCCTATCTCTGATACCGTTGTCC	TGCCGCAACTACTGTGATTCGG	145
Mouse-COL1A1	CCTCAGGGTATTGCTGGACAAC	CAGAAGGACCTTGTTTGCCAGG	115
Mouse-α-SMA	TGCTGACAGAGGCACCACTGAA	CAGTTGTACGTCCAGAGGCATAG	138
Mouse-MBNL1	CAAGCCTGGTTCCAGCAGAGAT	AATTGCCACGCTGGTACTCTCG	153
Human-GAPDH	GTCTCCTCTGACTTCAACAGCG	ACCACCCTGTTGCTGTAGCCAA	131
Human-FN1	CGGTGGCTGTCAGTCAAAG	AAACCTCGGCTTCCTCCATAA	130
Human-COL1A1	GTGCGATGACGTGATCTGTGA	CGGTGGTTTCTTGGTCGGT	119
Human-α-SMA	TGTGGCTATCCAGGCGGTGC	TCTCGGCCAGCCAGATCCAGAC	153
Human-MBNL1	GATCTTGCCGACTGCACCAATG	TTGCCACGTTGGTACTCTCGAC	132
Human-BAX	TCAGGATGCGTCCACCAAGAAG	TGTGTCCACGGCGGCAATCATC	103
Human-BCL2	ATCGCCCTGTGGATGACTGAGT	GCCAGGAGAAATCAAACAGAGGC	127

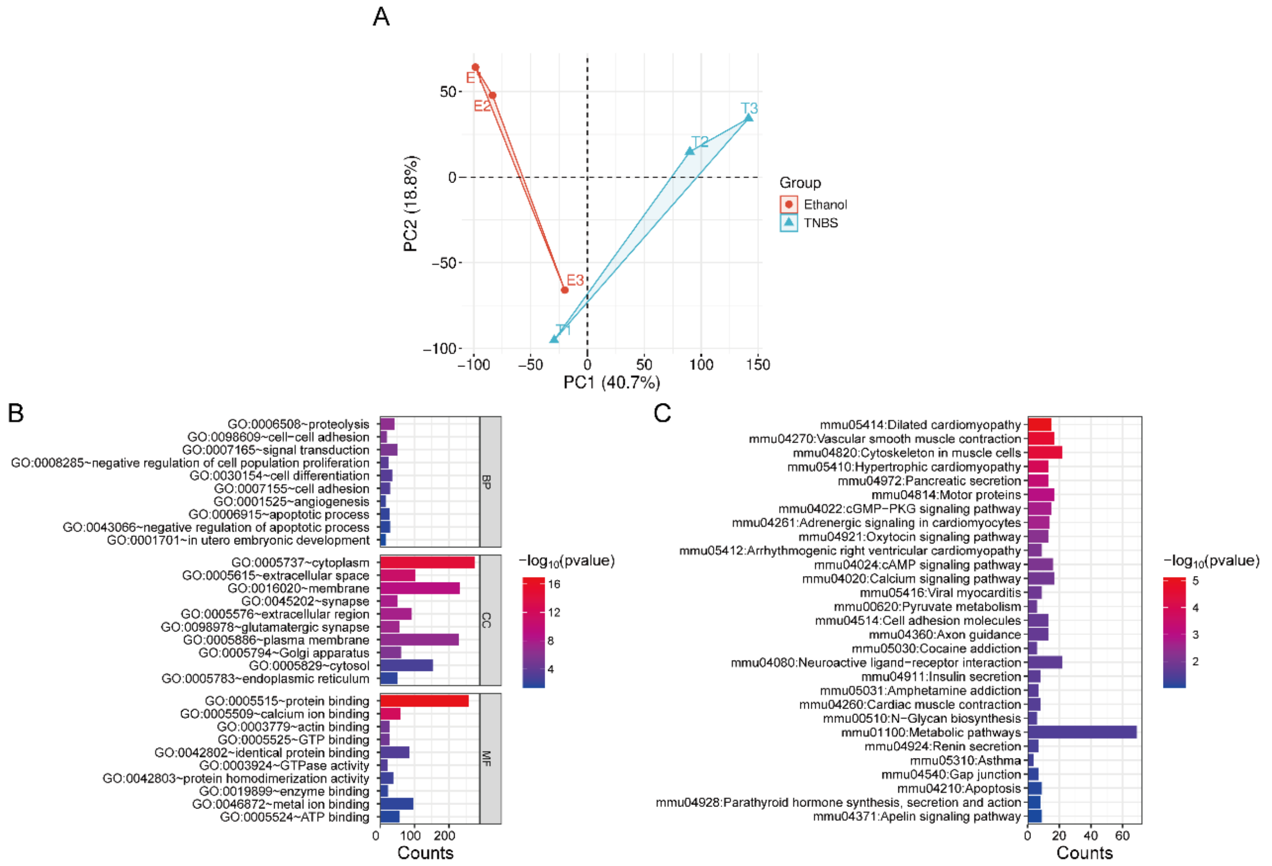

Figure S1. Transcriptome sequencing and bioinformatics analysis of mouse colon tissue. (A) Principal component analysis (PCA) of sequencing samples from Ethanol (red, n=3) and TNBS (blue, n=3) groups. (B) GO functional enrichment analysis of differentially expressed genes. (C) KEGG pathway enrichment analysis of differentially expressed genes.

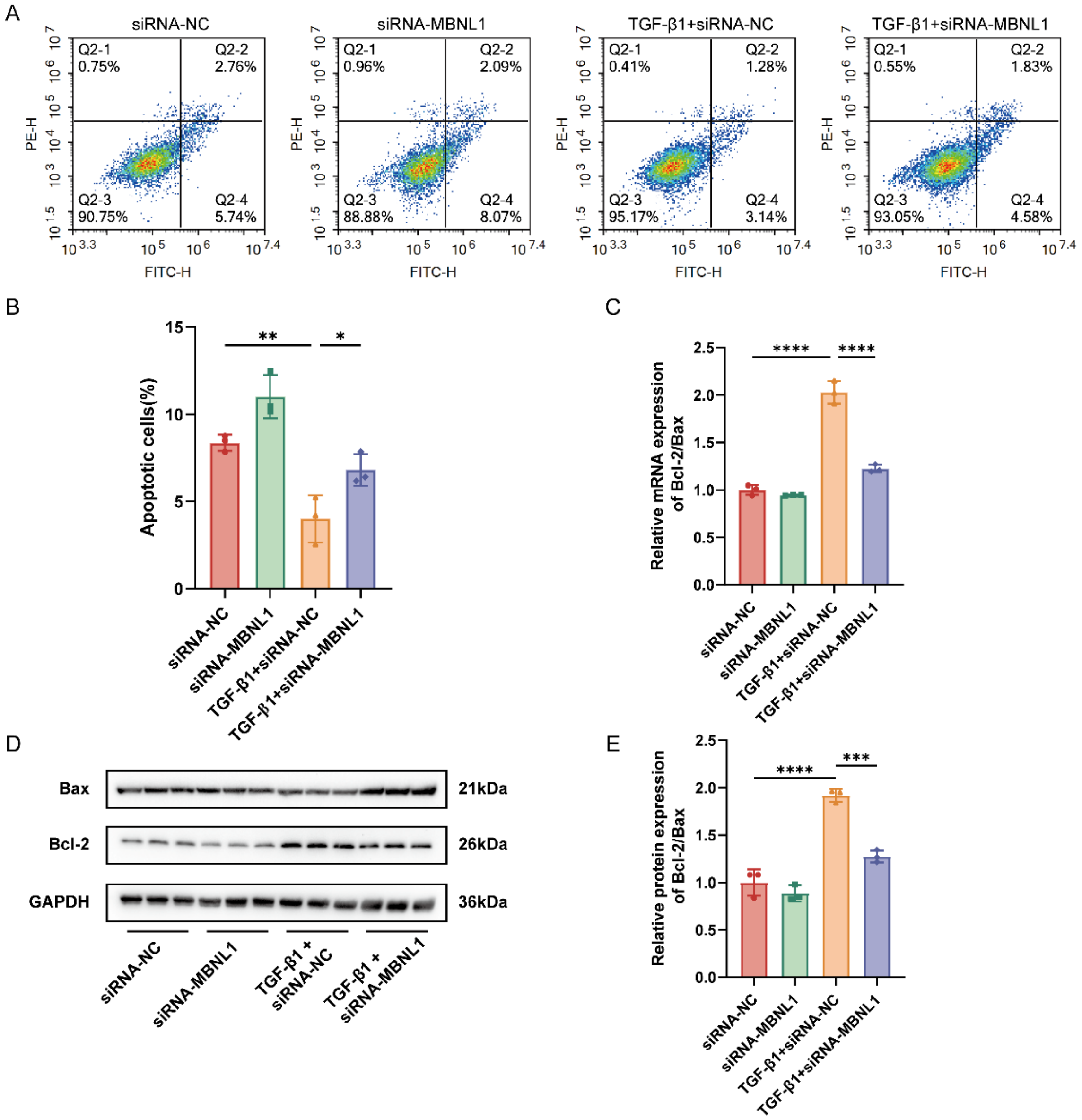

Figure S2. Effect of MBNL1 knockdown on apoptosis in CCD-18Co. (A) Representative flow cytometry dot plots (Annexin V/PI staining). (B) Apoptosis rate. (C) Relative mRNA expression levels of BCL-2 and BAX. (D) Representative Western blot bands of BCL-2 and BAX. (E) Relative protein expression levels of BCL-2/BAX ratio. Data are presented as mean ± SD (n=3). Comparisons among multiple groups were performed using one-way ANOVA followed by Tukey's post hoc test. ns, $P \geq 0.05$; *$P < 0.05$; **$P < 0.01$; ***$P < 0.001$; ****$P < 0.0001$.

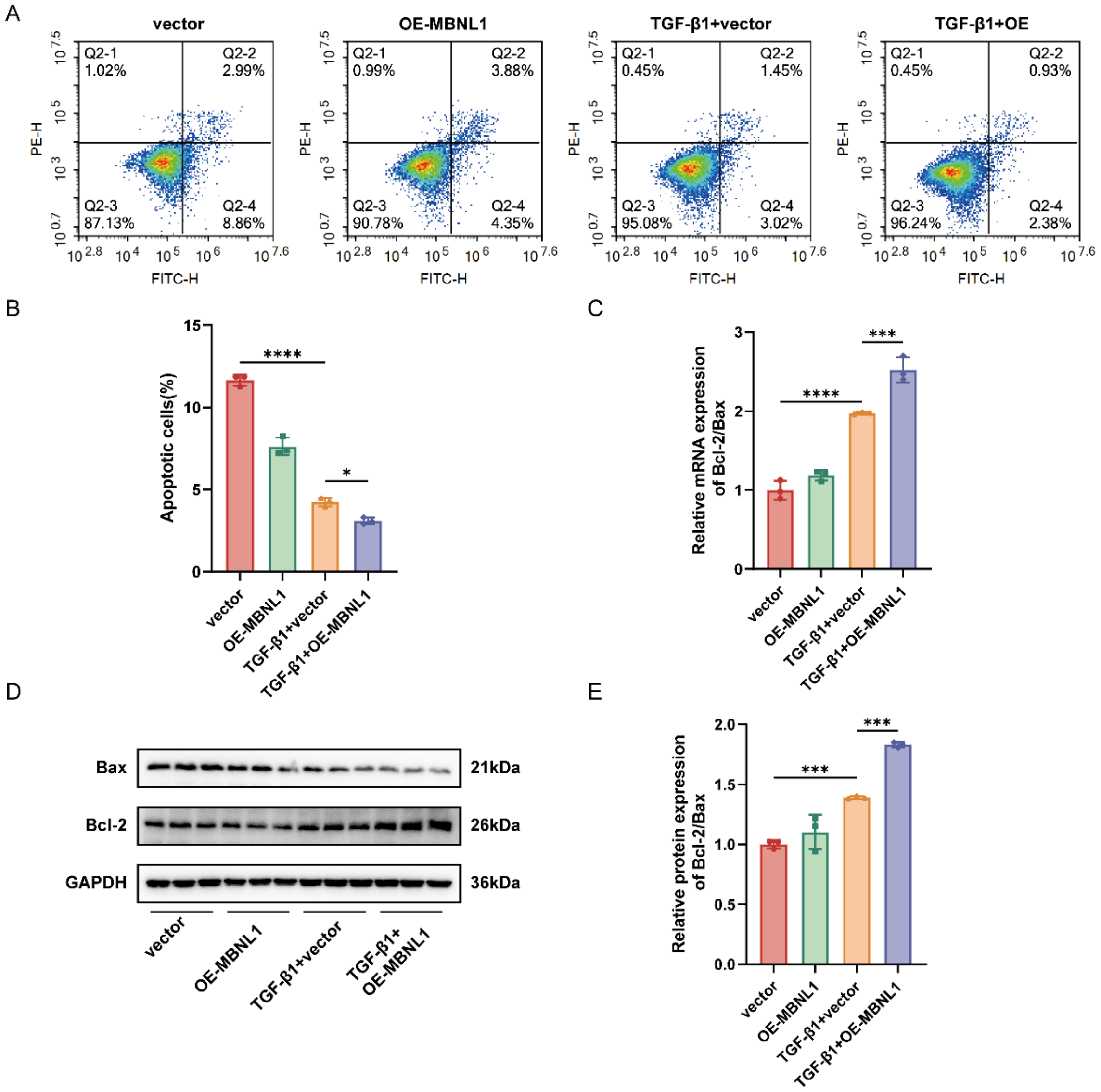

Figure S3. Effect of MBNL1 overexpression on apoptosis in CCD-18Co. (A) Representative flow cytometry dot plots (Annexin V/PI staining). (B) Apoptosis rate. (C) Relative mRNA expression levels of BCL-2 and BAX. (D) Representative Western blot bands of BCL-2 and BAX. (E) Relative protein expression levels of BCL-2/BAX ratio. Data are presented as mean ± SD (n=3). Comparisons among multiple groups were performed using one-way ANOVA followed by Tukey's post hoc test. ns, $P \geq 0.05$; $^{*}P < 0.05$; $^{**}P < 0.01$; $^{***}P < 0.001$; $^{****}P < 0.0001$.
